# Assessing Diet as a Modifiable Risk Factor for Pesticide Exposure

**DOI:** 10.3390/ijerph8061792

**Published:** 2011-05-25

**Authors:** Liza Oates, Marc Cohen

**Affiliations:** School of Health Science, RMIT University, PO Box 71, Bundoora, Victoria 3083, Australia; E-Mail: marc.cohen@rmit.edu.au

**Keywords:** pesticides, exposure assessment, agricultural exposures, organic diets, biomonitoring

## Abstract

The effects of pesticides on the general population, largely as a result of dietary exposure, are unclear. Adopting an organic diet appears to be an obvious solution for reducing dietary pesticide exposure and this is supported by biomonitoring studies in children. However, results of research into the effects of organic diets on pesticide exposure are difficult to interpret in light of the many complexities. Therefore future studies must be carefully designed. While biomonitoring can account for differences in overall exposure it cannot necessarily attribute the source. Due diligence must be given to appropriate selection of participants, target pesticides and analytical methods to ensure that the data generated will be both scientifically rigorous and clinically useful, while minimising the costs and difficulties associated with biomonitoring studies. Study design must also consider confounders such as the unpredictable nature of chemicals and inter- and intra-individual differences in exposure and other factors that might influence susceptibility to disease. Currently the most useful measures are non-specific urinary metabolites that measure a range of organophosphate and synthetic pyrethroid insecticides. These pesticides are in common use, frequently detected in population studies and may provide a broader overview of the impact of an organic diet on pesticide exposure than pesticide-specific metabolites. More population based studies are needed for comparative purposes and improvements in analytical methods are required before many other compounds can be considered for assessment.

## 1. Introduction

Pesticides are manufactured to be toxic to living organisms, but are not necessarily specific to their target species. They are deliberately released into the environment where their ubiquitous presence may endanger other living species, including humans [1]. It is unsurprising then that numerous published studies suggest a link between pesticide exposure and human health risks such as cancer [2], and adverse genotoxic, neurologic, and reproductive effects [3]. Obvious health risks may be due to acute poisoning or high level occupational exposure, while there is the possibility of more subtle health risks through general exposure via the food chain.

Globally around three million accidental or intentional pesticide poisonings occur each year resulting in around 260,000 deaths [4]. The vast majority occur in developing countries, which use only a fraction (20%) of the world’s agrochemicals [5]. However, these figures do not take into account chronic or cumulative health effects or effects arising from exposure during critical periods of development [6].

### 1.1. Occupational Exposure to Pesticides

There are numerous examples cited in the scientific literature regarding occupational exposure to pesticides and adverse health outcomes such as various cancers, Parkinson’s and other chronic diseases, as well as potential adverse effects on mental health and reproduction [7–12].

The United States Agricultural Health Study (AHS), a large prospective cohort study of pesticide applicators and their spouses, identified links between various pesticides and cancer incidence (lung, pancreatic, colon and rectal, all lymphohaematopoietic cancers, leukaemia, non-Hodgkin lymphoma, multiple myeloma, breast, bladder, prostate, brain, melanoma and childhood cancers). Outside the AHS, epidemiologic evidence remains limited with respect to many of these associations, but animal toxicity data support the biological plausibility of these relationships [7].

In addition to cancer, pesticides have been associated with a number of other health effects in animals and humans. The AHS has investigated conditions as widespread as Parkinson’s Disease, depression, diabetes, respiratory disorders and other health conditions [7]. Links to Parkinson’s Disease have been supported by experimental studies indicating that high exposure to paraquat (herbicide) and maneb (fungicide) may increase the risk in genetically susceptible individuals [8,9] highlighting concerns of potential epigenetic effects (gene-environment interactions). That a number of pesticides directly target the nervous system as their mechanism of toxicity may provide additional concerns. Studies in pesticide workers have also demonstrated effects on neurotransmitters which may be involved in mood regulation [10,11].

The risks of pesticide exposure at occupational levels may be of specific concern during critical developmental periods. Despite safeguards for pregnant farm workers, current measures may not be sufficient to protect the developing foetus from endocrine disrupting agents. For example a Danish study has reported that sons of women occupationally exposed to pesticides have a statistically significant decrease in penile length and a trend towards reduced testicular volume and serum concentrations of testosterone [12].

There are many uncertainties however, due to the limited number of research studies conducted on specific exposure-outcome relationships and methodological limitations such as crude exposure measurements, small sample sizes, and limited knowledge and control of potential confounders [13]. Furthermore, the sheer number of chemicals and variety of chemical actions involved, and the attribution of some adverse health effects to pesticides that are no longer in current-use in many regions make it extremely difficult to generalise about the health effects of pesticides.

### 1.2. Other Sources of Pesticide Exposure

While occupational exposure is likely to incur a greater risk, all humans are exposed to pesticides whether they be ingested from food sources, absorbed through the skin or inhaled from polluted air.

Dietary exposure from the ingestion of contaminated food (more so than water or other beverages) is considered to be the primary route of exposure for most pesticides although additional environmental exposure is also likely [14–16]. Food can be contaminated by pesticides used during production, transport or storage. While diet has been shown to be a significant predictor of pesticide exposure in all age groups, specific foods and food choices must also be considered as some foods may have a greater impact on exposure levels [17]. Food consumption patterns will vary among and within individuals for economic, seasonal, regional, cultural, ethical and personal reasons.

Non-dietary pesticide exposure can occur as a result of residential pesticide use (home, garden, pets, personal insect repellents), proximity to agricultural areas, time spent in parks and recreational areas or fumigated buildings, or hand to mouth activity (generally higher in young children). With the exception of residential use, most of these factors are outside the reasonable control of the average individual, whereas diet represents a modifiable risk factor that may be under individual control.

### 1.3. Monitoring Pesticide Exposure

Biological monitoring techniques (biomonitoring) assess pesticide levels in human tissue, and provide a measure of an individual’s total exposure to pesticides through dietary and non-dietary sources. Unfortunately biomonitoring data is not available for all pesticide classes or for all regions.

Some European countries [15], the CDC in the USA [18], and Health Canada [19] have conducted large scale biomonitoring studies assessing pesticide exposure in the general population, although such studies have not been conducted in countries such as Australia or most developing countries. These studies frequently detect pesticides or their metabolites in human tissue. The mean levels are almost always lower than those found in occupationally exposed individuals although those in the higher range can be similar to some occupationally exposed workers [15]. As the half-lives of modern pesticides are very short (often <24 hours), these data suggest that the population is continually and routinely exposed to pesticides [15].

### 1.4. Non-Occupational Exposure to Pesticides

Identifying health risks in non-occupationally exposed populations is difficult as pesticide exposure is diffuse and the source of exposure (dietary, environmental *etc*.) is not always clear. Of particular concern is the increased risk associated with pesticide exposure during critical periods of development, such as preconception, prenatal and early childhood. For example, high urinary levels of atrazine, alachlor and diazinon have been associated with abnormal sperm [20]. In the US a significant association has been reported between the months of increased risk of a birth defect and increased levels of pesticides (especially atrazine) in surface water [21]. Higher prenatal urinary concentrations of dialkyl phosphate (DAP) (which are metabolites of organophosphate pesticides [OPs]) have been associated with poorer intellectual development in 7-year-old children [22] and elevated levels of DAPs have also been associated with an increase in the prevalence of ADHD in children aged 8 to 15 years [23]. These DAP concentrations were within the range of levels measured in the general U.S. population although the reasons for these elevated levels are not clear.

In recent times there has been considerable media attention around obesity and insulin resistance. These are common conditions which can influence other disease processes and impact on quality of life and mortality. In rats chronic administration of low concentrations of atrazine has been shown to increase body weight, intra-abdominal fat and insulin resistance and reduce basal metabolic rate. While obesity and insulin resistance were further exacerbated by a high-fat diet they also occurred without changing food intake or physical activity level [24]. Adding to these concerns, data from the Center for Disease Control and Prevention (CDC) shows an apparent overlap between areas of heavy atrazine use in the USA and the prevalence obesity (BMI > 30) [25].

As the primary route of exposure for most pesticides is via the ingestion of food exposed through conventional agricultural practices [14–16], such findings in addition to uncertainly about the evaluation of pesticides [26], raise concern amongst some consumers.

## 2. Organic Diets as an Intervention

Organic farming practices do not use synthetic pesticides and data from food residue surveys confirm that organic produce has reduced pesticide levels [27–29]. This provides a rationale that organic food consumption should result in reduced pesticide exposure. However, studies describing reduced risk of developing pesticide related diseases, or improved health outcomes as a result of consuming organic foods are lacking. Despite a lack of supporting research, adopting an ‘organic diet’ appears to be an obvious way to reduce pesticide exposure for a growing number of concerned individuals. Some believe that ‘on the basis of the precautionary principle alone, choosing organic food appears to be an entirely rational decision’ [30]. Assessing the efficacy of such an intervention, however, is not a simple feat.

In a recent attempt initiated by the Food Standards Authority (FSA) in the UK to investigate the ‘putative health effects’ of organic food, studies that were primarily concerned with chemical residues (including pesticides) were specifically excluded. The focus on nutrition-related health effects yielded only twelve relevant articles [31]. In one study the consumption of organic dairy products within the context of a general organic diet was associated with a 36% lower risk of infantile eczema in children who exclusively consumed organic dairy products (*i.e.*, weaned on organic milk, cheese and yoghurts and who were breastfed by mothers eating organic dairy products). However, the authors attributed these results to increased levels of omega-3 fatty acids and conjugated linoleic acid in organic compared to conventional milk and the likely reduction in pesticide exposure was not discussed [32].

Understanding the health impact of dietary pesticide exposure, and therefore any potential benefit of reducing exposure by adopting an organic diet, begins with determining actual exposure levels. While monitoring of pesticide residues in food may provide a useful insight into the potential sources of dietary exposure, biomonitoring is more likely to correlate with adverse health effects as it directly measures the amount of a pesticide (or its metabolites or degradation products) in human tissue. However, it should be stated that high levels of these markers have not been consistently associated with adverse health effects [15].

Regarding organic consumers only a few published reports in children have utilised biomonitoring [33–35]. These have examined urinary metabolites of OP and synthetic pyrethroid insecticides (PYRs). Dietary exposure to other classes of pesticides such as carbamate insecticides; fungicides and herbicides has not been formally evaluated in organic consumers.

In 2003 Curl *et al*. reported that children who consumed organic fruit, vegetables and juice had a mean total urinary dimethyl alkylphosphate metabolite (DMAP) concentration (a non-specific measure of OP exposure) that was approximately nine times lower than children consuming conventional foods. This corresponded to a reduction in the children’s exposure levels from above to below the U.S. Environmental Protection Agency’s guidelines, shifting exposures from a range of uncertain risk to negligible risk [33].

The results of the Curl study are supported by the Children’s Pesticide Exposure Study (CPES) [34] which also reported reductions in urinary pesticide metabolites in children consuming organic produce. This study included measurements of select urinary OP and PYR metabolites in 23 children aged 3–11 years over a 15-consecutive-day sampling period. Children consumed their usual conventional diet with an organic intervention phase for five consecutive days, at which time organic food items were substituted for most of the children’s conventional diet (fruit, vegetables, juice, wheat and corn products). The organic intervention resulted in a decrease in certain pesticide-specific OP metabolites to non-detectable or close to non-detectable levels [14] and a reduction of approximately 50% in PYR exposure [35]. These results confirm that consumption of organic produce appears to provide a relatively simple way to reduce children’s exposure, especially to OP pesticides [14,33], and that this occurs relatively quickly. However, drawing any general conclusions from these biomonitoring studies to support the hypothesis that organic diets reduce pesticide exposure will require further studies in different population groups.

## 3. Complexities and Limitations of Biomonitoring

Designing biomonitoring studies to assess the efficacy of an organic diet in reducing pesticide exposure must be carefully devised. Appropriate study design requires consideration of the limitations of biomonitoring and the complexities involved in contextualising the results. This includes careful selection of which pesticides will be targeted and the most appropriate analytical methods to use. Ideally the methods chosen should be able to attribute the source of exposure to dietary intake. Study design must also consider confounders such as the unpredictable nature of chemicals and individual genetic and environmental factors that might influence susceptibility to disease. Contextualising the results also requires consideration of the data available for comparison purposes.

### 3.1. Study Design

The population of interest needs to be clearly defined with careful consideration of factors that may affect exposure and susceptibility. Consideration should be given to whether the study will use an organic intervention or observe free living organic consumers eating their usual diet. As free-living consumers are unlikely to consume a 100% organic diet detailed survey instruments need to record dietary intake to quantify the level of organic consumption. Other sources of exposure and potential confounding factors such as age, health status, medication use and other factors that may influence the metabolism of, and susceptibility to, pesticides must also be determined.

Targeted pesticides need to be selected based upon the likelihood of dietary exposure in the general public. This is likely to vary from region to region and over time depending on prevalence of use but may be informed by studies which monitor pesticide residues in food. Seasonal and regional variations can be anticipated depending on the time of the year and the nature of pest infestations. Priority might be given to assessment of pesticides with high prevalence of use, those with the greatest public health concerns or to newer chemicals so that potential human health risks can be more accurately determined. Once chosen the most appropriate methods of testing these pesticides must be considered.

### 3.2. Analytical Methods

There is an increasing amount of biomonitoring data available and Barr [36] and Aprea *et al*. [1] have previously described biomonitoring methods for assessing pesticide exposure. However choosing and conducting such tests requires a high level of technical expertise. Scientists do not always agree on the most appropriate methods for assessing pesticide exposure, limits of detection may vary and data collection and analysis can be laborious, expensive and place unacceptable demands on study participants.

In humans, most current-use pesticides are excreted within 24 hours as either the parent pesticide, a mercapturic acid detoxification product or as a metabolite [36]. Therefore collecting samples that degrade quickly requires a level of urgency. While many herbicide compounds are poorly metabolised and are excreted largely unchanged in the urine [1], the parent compounds of many other pesticides are metabolised very rapidly, making their measurement impractical. As a result, metabolites are often used as surrogate markers for exposure. Several methods have been reported which measure intact OP pesticides in blood, serum, or plasma, however, for the most part these tests are used for detecting acute poisoning or very high levels of exposure [37]. Similarly, occupational exposure to PYRs can be assessed by monitoring intact PYRs, yet due to their rapid elimination, unmodified compounds are less sensitive indicators of exposure than the metabolites [1] and thus may not be suitable for detecting differences in dietary exposure.

Determining the most appropriate tests is not always straightforward. For example according to Barr [36], atrazine mercapture is often tested but may not be the best marker for atrazine exposure, recommending instead analysis of dealkylated or hydroxylated metabolites of triazine herbicides, mercapturic acids of the dealkylation products or free atrazine. Determining suitable limits of detection (LODs) may also be open to conjecture. As it is difficult to confidently determine the levels of pesticide exposure that are safe under all circumstances [26], the LODs should be set as low as possible. Lower detection limits will yield more samples with detectable metabolites, and lower LODs will more accurately reveal differences in dietary exposure between consumers of organic and conventional food [15].

Defining appropriate sampling times and collecting representative samples can be difficult; and pure standards for measuring pesticide metabolites are not always available [1]. Analytical methods often involve gas chromatography (GC) or high-performance liquid chromatography (HPLC) following sample preparation and extraction requiring specialised equipment and technicians. The choice of analytical methods must also consider practicalities such as financial restraints and the potential burden on study participants and researchers. This may include whether invasive methods are required to collect samples and the timing and costs of such procedures.

### 3.3. Attributing the Source of Exposure

Although useful in determining an individual’s total exposure (dietary and non-dietary) to pesticides, biomonitoring methods are not always able to attribute the source of exposure, especially when metabolites are used. Metabolites may reflect exposure to more than one parent pesticide, may be markers for substances other than pesticides, or may be preformed or result from biological processes in the body.

Some metabolites are markers for specific pesticides while others are representative of a number of pesticides. Urinary 3-phenoxybenzoic acid (3PBA) is a non-specific metabolite common to a number of PYRs, and *trans*-3-(2,2-dichlorovinyl)-2,2-dimethylcyclopropane carboxylic acid (*trans*-DCCA), is common to permethrin, cypermethrin, and cyfluthrin [14]. With OPs the most commonly reported method is to measure DAP metabolites which are formed in the human body during the metabolism of OP pesticides and excreted in urine [18]. The data generated can provide a cumulative index of exposure to most members of the OP class but are not pesticide-specific. Each DAP metabolite is associated with a number of OPs, and many OPs can form more than one of these metabolites [37]. Specific biomarkers for individual pesticides in this class are also available, such as 3,5,6-trichloro-2-pyridinol (TCPy) for chlorpyrifos and malathion dicarboxylic acid (MDA) for malathion. However urinary DAPs may provide a more useful assessment for exposure to the class in general and this may be advantageous in providing an overview of the impact of an organic diet. If the purpose however, is to determine the effect of the diet on individual pesticides then pesticide-specific markers may be more useful.

Some metabolites utilised in biomonitoring studies are not entirely specific to pesticides. For instance, 1-naphthol (1NAP), a metabolite of carbaryl is also a marker for the ubiquitous naphthalene (found in mothballs, petroleum and cigarette smoke) [15]. A further consideration is the potential contribution from preformed metabolites. This can occur with OP metabolites such as DAPs which may be detected as a result of the metabolism of ingested parent compounds but may also result from the ingestion of preformed metabolites which may be present on food as a result of environmental degradation. In addition sources of inorganic phosphate may be alkylated within the body to form dimethylphosphate (DMP), and this may also contribute to urinary DAP levels [15].

### 3.4. Unpredictable Nature of Chemicals

When attempting to understand the impact of individual pesticides on human health, consideration must be given not only to the specific chemicals targeted in the biomonitoring study but also to the potential impact of other chemicals and risk factors for disease progression. We have previously described some of these factors including: the effects of exposure to mixtures of chemicals; the dose, duration and timing of exposure; the complexities and lack of complete safety assessment data; as well as variations in the exposure, metabolism and susceptibility of different individuals [38].

Humans are exposed to a unique and ever changing cocktail of chemicals. This cocktail may include pesticides and other chemicals acquired through ingestion, inhalation or dermal absorption. Some of these substances may have similar mechanisms of action or may interact via toxicokinetic (absorption, distribution, metabolism and excretion) or toxicodynamic (binding, interaction and induction of toxicity) processes to produce additive, antagonistic or synergistic effects [39]. For instance the synergistic effects of mixtures of sub-lethal doses of OPs in juvenille salmon are sufficient to cause anticholinesterase intoxication and death [40].

Although most pesticide formulations are mixtures of chemicals, most safety assessment methods focus on individual ‘active’ chemicals rather than ‘whole formulations’ including their adjuvants, metabolites and degradation products. A case in point is glyphosate. The adverse effects associated with glyphosate appear to be more dependent on the formulation tested than on the glyphosate concentration [41,42]. It is possible that these effects may be more appropriately attributed to other compounds in the formulation or to the environmental breakdown product of glyphosate, aminomethyl phosphonic acid (AMPA) [42-44]. Similarly recent studies suggest that prenatal exposure to piperonyl butoxide (a PYR synergist) has been negatively associated with neurodevelopment [45].

Depending on the disease process in question non-chemical risk factors such as physical inactivity or nutrient deficiencies or excesses as well differences in genetic susceptibility, may also confound results. Using biomonitoring data from a few select targeted chemicals is unlikely to provide sufficient data to deal with the inherent complexities of disease progression.

### 3.5. Individual Factors

In addition to chemicals behaving in potentially unpredictable ways, an individual’s response to chemicals may also be unpredictable. Although a 100-fold safety factor is taken into account when establishing acceptable daily intakes for humans [26], this must overcome differences between experimental and real world conditions, as well as account for individual variability in exposure and metabolism. There is currently insufficient data from epidemiological studies to confidently predict the levels of pesticides (either the parent compounds, metabolites, degradation products or adjuvants) that might be associated with human health risks and such levels are likely to be highly variable. For example levels of 3PBA are known to be influenced by factors such as tobacco use, time spent gardening and the use of cytochrome p450-inhibiting medications [17]. This may in part reflect differences in exposure but also differences in the metabolism of pesticides and these are likely to vary not only between different individuals but also within the same person over the course of their lifetime. A progressive increase in DMAP metabolites at 6, 12 and 24 months of age has been positively associated with the number of children’s daily servings of fruits and vegetables [46]. At the same time the activity of enzymes which play an important role in the detoxification of many pesticides are known to be impaired in children [47].

To a limited extent biomonitoring can account for poorly understood processes such as bioaccumulation, excretion and metabolism [37], but demonstrating pesticide exposure at a specific time point does not provide information about the lifetime exposure to pesticides or the increased risk of exposure during critical periods of development (such as *in utero*). Assessing risk relies not only on determining individual exposure but must also consider variations in an individual’s ability to metabolise, detoxify and excrete mixtures of chemicals as well as their susceptibility to disease which may vary with genetic, developmental, physiological and environmental conditions.

### 3.6. Comparative Data

Once measurements have been collected the results must be carefully interpreted. Where possible, results from organic consumers may be compared with reference values of the general population although such studies do not enquire about levels of organic food consumption [1].

OPs are frequently detected in general population studies [15,18,19] and have been assessed in comparative studies of children consuming organic and conventional diets [14,33]. In the CPES the pesticide-specific OP metabolites TCPy and MDA had the highest frequency of detection representing chlorpyrifos and malathion exposure from the conventional diet [14]. PYR metabolites have also been detected with varying frequency in population studies [15,18,19] and differences have been observed in children when switched from a conventional diet to an organic diet for 5 days [48].

For both general population and organic consumption studies there may be significant heterogeneity with regard to the pesticides chosen for monitoring and the methods and LODs used. Methods and detection performance have improved over time, especially for OP metabolites, so care must be taken when attempting to compare results from older studies [15].

## 4. Conclusions

The effects of pesticides on the general population, largely as a result of dietary exposure, are unclear. If the precautionary principle is applied then adopting an organic diet appears to be an obvious solution for reducing pesticide exposure and this is supported by biomonitoring studies in children. However the few attempts that have been made to determine the efficacy of such an intervention are difficult to interpret in light of the many complexities.

Biomonitoring cannot be considered an end in itself but simply a tool for integrated health assessment; an intermediate step for establishing a link between exposure and adverse health effects. The limitations of biomonitoring and the complexities involved in interpreting the results must be acknowledged. As previously mentioned, both dietary and non-dietary sources of exposure can vary among individuals. While biomonitoring can account for differences in overall exposure it cannot necessarily attribute the source. Due diligence must be given to appropriate study design and selection of analytical methods to ensure that the data generated will be both scientifically rigorous and clinically useful, while minimising the costs and difficulties associated with biomonitoring studies. Currently the most useful candidates for assessment are urinary DAPs and urinary 3PBA and *trans*-DCCA. These assessments provide evidence of exposure to OP and PYR insecticides respectively and as they are in common use they can provide a broader overview of the impact of an organic diet on pesticide exposure than pesticide-specific metabolites. As previously discussed these metabolites have frequently been detected in population studies and have been assessed in children consuming organic foods providing useful data for comparison. However the contribution of preformed metabolites in the diet must be considered.

Depending on the prevalence of use in the region of interest, specific metabolites for chlorpyrifos (TCPy) and malathion (MDA) may also be incorporated. In addition select herbicides may be useful, although comparative data from similar studies is not currently available and the frequency of detection in population studies tends to be relatively low.

Despite its limitations, biomonitoring remains the most useful surrogate indicator of pesticide exposure currently available. The above discussion highlights some of the many issues encountered when selecting biomonitoring methods for assessment of pesticide exposure. It provides an outline of some of the complexities encountered when attempting to ascertain the efficacy of an organic diet intervention in reducing such exposure.
